# 

**Published:** 1917-09-08

**Authors:** 


### The Book Market.

LIST OF NEW BOOKS RECEIVED FROM THE FOLLOWING PUBLISHERS :—

H. K. Lewis and Co., Ltd. : "Electro-Therapeutics for Military Hospitals." By Wilfrid Garton, M.R.C.S., L.R.C.P. 2s. 6d. net. "Hygiene and Public Health." By Lewis C. Parkes, M.D., and Henry R. Kennard, M.B. 14s. net. "The Indian Operation of Couching for Cataract." B. R. H. Elliott, M.D., B.S. 7s. 6d. net.

The Mental Culture Enterprise, 329 High Holborn: "The Ideal Nurse." By Chas. Mercier. 1s. 3d. net. "The Principles of Rational Education." By Chas. Mercier, M.D., F.R.C.P. 2s. 9d. net.

### TO OUR READERS.

Contributions are specially invited from any of the readers of The Hospital. They should deal with topical subjects and news or incidents illustrating any department of hospital work, its progress or difficulties. They must be authenticated for the information of the Editor only. The minimum payment if published is 5s. There is no hard-and-fast rule as to space, but paragraphs of about twenty lines in length are preferred.

### MATRONS, SISTERS, AND NURSES.

☛ It is our invariable practice to pay for all items of news which we may publish. The minimum payment is 5s. Paragraphs of about twenty lines in length—that is to say, of 150 words or less—are preferred. Contributions should be addressed to The Editor, The Hospital, 28 and 29 Southampton Street, Strand, London, W.C., and should reach him by the first post on Mondays.

### Manager's Notices.

**Now Ready, "The Hospital" Index to Vol. LXI., Oct. 1916 to April 1917. Price 2½d. per copy post free.**

**All letters on business matters must be addressed to The Manager, "The Hospital," 28 and 29 Southampton Street, London, W.C. 2.**

**Subscriptions**, which may commence at any time, are payable in advance. Cheques and Post-Office Orders should be crossed London County and Westminster Bank, Covent Garden Branch, and made payable to the Manager of THE HOSPITAL.

RATES OF SUBSCRIPTION (payable in advance).

| | | |
|---|---|---|
| UNITED KINGDOM. | | |
| Three Months | (including Postage) | 2s. 0d. |
| Six Months | do. | 4s. 0d. |
| Twelve Months | do. | 6s. 6d. |
| FOREIGN AND COLONIAL. | | |
| Three Months | (including Postage) | 2s. 6d. |
| Six Months | do. | 5s. 0d. |
| Twelve Months | do. | 8s. 8d. |

**Advertisements:**

To ensure insertion, all advertisements for THE HOSPITAL must reach the Manager not later than Wednesday at noon in each case.

*SUBSCRIPTION FORM.*

*Please send me* THE HOSPITAL *for* .............. *months, commencing with your issue of* ........................., *for which please find enclosed* ........................

*Name* ..........................................

*Address* ..........................................

..........................................

*Date* ..........................................

*To* .......................................... *Newsagent.*

### Publications. *Continued on p. iii.*

NEW EDITION in Two Vols. 45s. net.

**DEFORMITIES, INCLUDING DISEASES of the BONES and JOINTS.**

A Text-Book of Orthopædic Surgery.

Illustrated by 70 Plates and over 1,000 Figures and by Notes of Cases.

By A. H. TUBBY, M.S.Lond., F.R.C.S.Eng.

"The standard text-book in English on the subject."—*Lancet.*
First Edition. "Standard work on the subject in the English language." *British Medical Journal.*

MACMILLAN & CO., LTD., St. Martin Street, London, W.C.

Now Ready. Third Edition, Revised and Enlarged. Price 3s. 6d. net.

**INSANITY IN EVERYDAY PRACTICE.**

By E. G. YOUNGER, M.D., M.R.C.P., D.P.H.

London: BAILLIÈRE, TINDALL & COX, 8 Henrietta Street, Covent Garden.

A LIST of the NEW PUBLICATIONS of THE SCIENTIFIC PRESS, LIMITED, will be sent post free to any member of the Medical Profession on receipt of post card.

28 & 29 SOUTHAMPTON STREET, STRAND, LONDON, W.C.

FOR THE PROFESSIONAL NURSE.

*THIRD EDITION. Price 3s. 9d. post free.*

**SICKROOM COOKERY AND HOSPITAL DIET,**

with Special Recipes for Convalescent & Diabetic Patients.
By MAUDE EARLE.

With NOTES ON THE FEEDING OF INFANTS.

LONDON: SPOTTISWOODE, BALLANTYNE & CO. LTD., 1 New-street Square, E.C. 4.

**AMBIDEXTERITY AND MENTAL CULTURE.**
*By H. Macnaughton Jones, M.D., M.Ch., F.R.C.S. I. & Ed.*
Fcp. 8vo. 2s. 6d. net.
The subject of this volume has recently attracted the attention of those interested in the training of the young, and is also the object of careful study by neurologists and physiologists. This book summarises all the latest information.
"*A most powerful & convincing plea for the use of both hands equally.*"—GLOBE.
LONDON WM. HEINEMANN.

